# Developing a CBT-Based Intervention Program for Reducing School Burnout and Investigating Its Effectiveness With Mixed Methods Research

**DOI:** 10.3389/fpsyg.2022.884912

**Published:** 2022-07-18

**Authors:** Sümeyye Ulaş, İsmaİl Seçer

**Affiliations:** Department of Psychological Counseling and Guidance, Atatürk University, Erzurum, Turkey

**Keywords:** school burnout, cognitive-behavioral therapy, psychoeducation, mixed methods research, secondary school students (adolescents)

## Abstract

This study sets out to develop a cognitive-behavioral therapy-based psychoeducation application to reduce the school burnout levels of secondary school students and to test its effectiveness with the mixed methods research design. For this purpose, qualitative data have been included in the process at three different steps, before, during, and after the experimental application involving an intervention application. The application of the intervention of the research has been carried out with an experimental design with pretest-posttest control group, which is one of the true experimental designs. Experimental and control groups have been determined with 30 students reached by nested sampling method, and the CBT-based psychoeducation practice developed during the research process has been carried out for 9 weeks in the experimental group. During the research, descriptive analysis and content analysis methods have been used in the analysis of qualitative data, normality analysis, and One-Way Analysis of Covariance have been used in the analysis of quantitative data. Findings obtained from the study show that CBT-based psychoeducation practice is an effective approach in reducing school burnout. It has also been determined that the findings obtained from the analysis of the documents obtained during the application and findings from the interview process done after the application coincided with the findings of quantitative methods, and the qualitative findings adequately explain the quantitative findings.

## Introduction

School life has a unique quality in that it provides students with the environment and acquisitions they will need academically, socially, and emotionally (Wilson and Tanner-Smith, 2013; Kearney and Graczyk, 2014). On the other hand, problems such as school burnout, which arise due to both school-related and personal characteristics (Alarcon et al., 2011; Romano et al., 2020), limit students’ acquiring those and using the facilities provided by the school (Seçer, 2015a). Although the concept of burnout is predominantly a problem area related to professional life (Maslach and Leiter, 1997), it has started to be associated with similar symptoms observed in students in recent years (Misra and McKean, 2000; Salmela-Aro et al., 2009b; Seçer et al., 2013; Romano et al., 2020). Although being a student is not a job or a profession, it can be thought of as a “job” because its academic duties and responsibilities continue for many years and this process forces the cognitive, emotional and even physical resources of children (Salmela-Aro et al., 2009b). Children have many responsibilities such as attending school, doing homework and passing exams for most of the year. In addition, they are faced with the phenomenon of fulfilling the relatively high demands and expectations of their parents and teachers (Seçer, 2015b). Children may develop chronic responses such as being exhausted, developing negative attitudes toward school and school activities, and feeling inadequate (Schaufeli et al., 2002; Lee et al., 2010), decrease in academic achievement (Zhang et al., 2007) and losing ability to cope with the difficulties of this process (Romano et al., 2020) because of this ongoing responsibility and high expectations. These symptoms, which become chronic, also put pressure on the mental health of children (Misra and McKean, 2000; Gil-Monte, 2005; Seçer, 2015a). Thus, children may develop a meaningless and cynical affection toward school and eventually lose their satisfaction by seeing themselves as inadequate, useless and unsuccessful as a student (Salmela-Aro et al., 2009b; Walburg, 2014).

School burnout, which turns into an important pressure tool on social, emotional and physical health, is a problematic that consumes students’ available resources (Alarcon et al., 2011; Seçer, 2015b) and when it is not prevented, it has long-term and important consequences. It is argued that school burnout will cause secondary problems such as deterioration in social skills and interpersonal relationships (Yang and Farn, 2005), lack of empathy and health problems (Dyrbye et al., 2012; Mazurkiewicz et al., 2012), depression and anxiety symptoms (Salmela-Aro and Parker, 2011; Seçer, 2015a), sleep disorders, substance use and suicidal ideation (Nteveros et al., 2020) etc. as well as school problems such as decrease in academic achievement and dropping out of school. Therefore, it can be said that school burnout is an important mental health problem that threatens the students academically, socially and emotionally, and it is clear that if it cannot be prevented, it will have long-term and important consequences. So, it is thought that students’ positions need to be strengthened in order to effectively deal with problems such as school burnout throughout their educational life (Aypay, 2017; Seçer and Ulaş, 2020). Although the negative impact of school burnout on children’s social, emotional and physical health has been clearly demonstrated (Salmela-Aro et al., 2009b; Alarcon et al., 2011; Walburg, 2014; Seçer, 2015b), it is considered that the majority of the studies in the literature do not go beyond cross-sectional/survey nature and therefore contain an important limitation. In the literature, no intervention-based study has been found, and there are various model suggestions. Aypay (2017) suggested that practices that strengthen subjective well-being and future attitudes in young people can contribute to the prevention of school burnout. Romano et al. (2020) suggested that school burnout can be prevented by increasing emotional intelligence and teacher support and through reducing academic anxiety. It is considered that developing and implementing intervention practices for school burnout from the early stages of school life will have an important protective function in terms of children’s mental health in the short and long term, and in this way, personal and academic achievements will be strengthened.

In this direction, Cognitive-Behavioral Therapy (CBT) is a frequently preferred approach in terms of intervention for various mental health problems related to adolescence. Along with the studies, it has been scientifically proven that cognitive-behavioral therapy is an effective approach to depression, anxiety, and avoidance in adolescence (Barrett, 1998; Verduyn, 2000; Kendall and Peterman, 2015; Rooksby et al., 2015). Ginsburg et al. (2008) found that a school-based CBT program was effective on anxiety. It has been found that CBT is an effective approach for improving social problem-solving skills in adolescents with behavioral problems (Matthys and Schutter, 2022), emotion regulation skills (Howells, 2018), and at the point of intervention in anxiety disorders in the context of anxiety sensitivity and emotion regulation (Asnaani et al., 2020). Hannan et al. (2019), on the other hand, evaluated that intensified CBT application in the intervention of school refusal, which is one of the school attendance problems, is promising in terms of increasing the time spent at school. In this context, the main purpose of the study was to develop a CBT-based intervention program based on the conceptual framework of school burnout and to test the effectiveness of secondary school students in reducing school burnout symptoms. According to this purpose, the research is based on the following questions and hypotheses.

*Research question:* “How do the qualitative findings obtained during the intervention (documents obtained from the students) and after the intervention (from the interviews) help in terms of explaining the quantitative findings of the intervention practice aimed at testing the psychoeducation program developed based on the interviews with secondary school students and the document analysis on school burnout?”

*Research hypothesis:* Psychoeducation application developed based on CBT is an effective approach in reducing school burnout in secondary school students.

The aims, questions and hypotheses of the research given above were designed with a mixed methods research process. In this direction, different qualitative and quantitative processes and stages were used simultaneously, sequentially and integrated in the process of both developing an intervention program and testing the effectiveness of the experimental procedure applied.

## Materials and Methods

### Research Design

**Figure 1:**
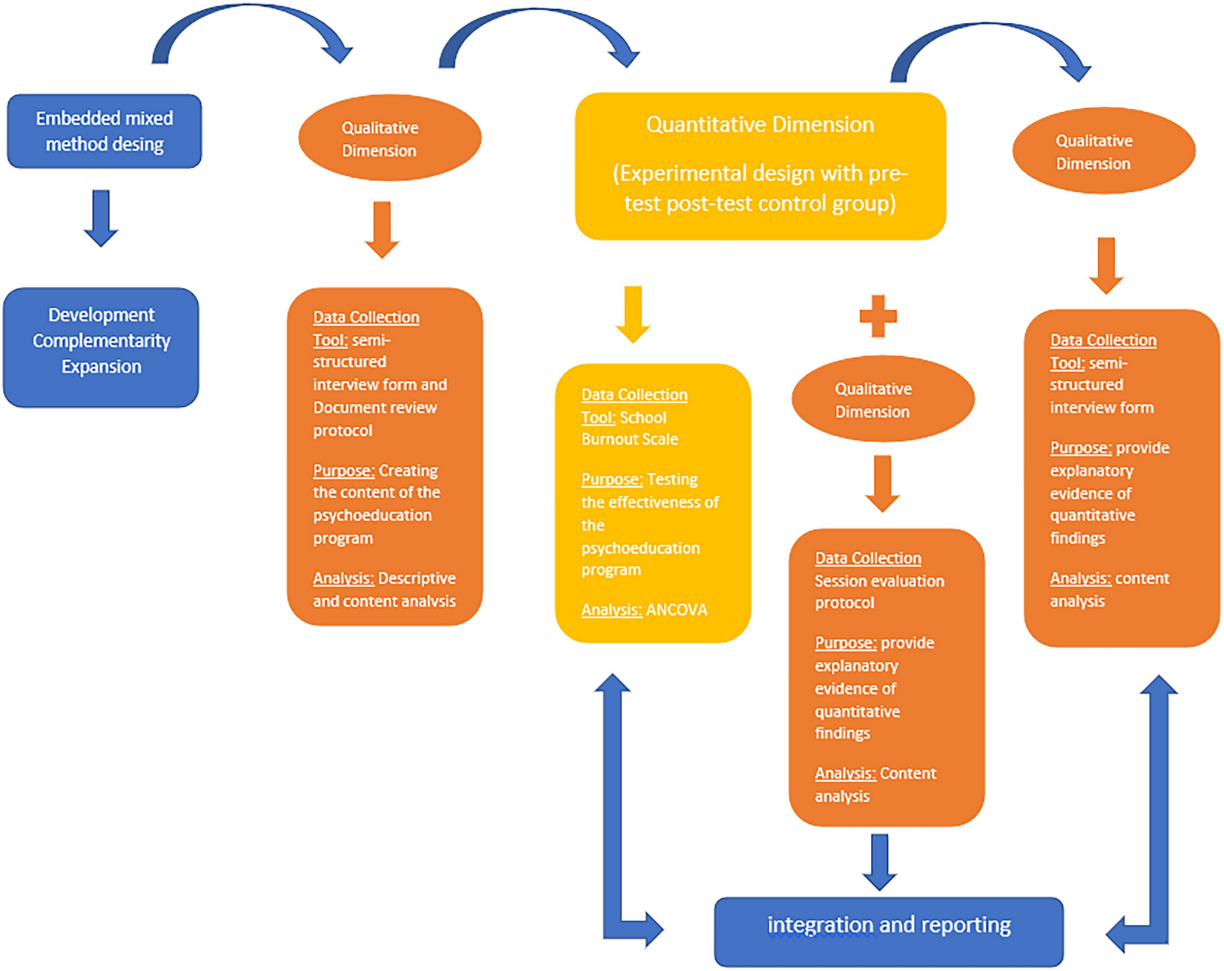
Research design.

**Figure 2:**
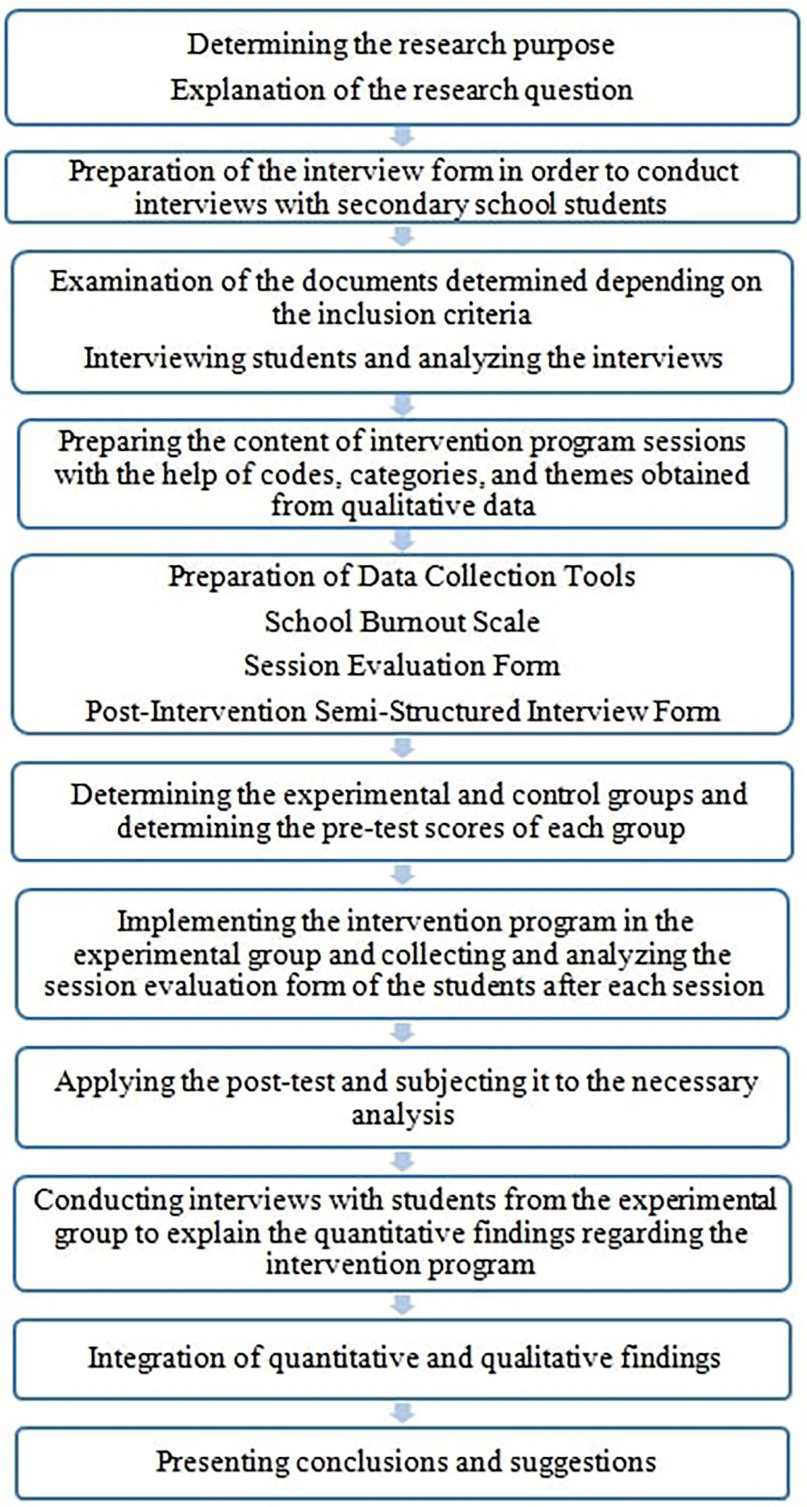
Research steps.

This study is a fully integrated mixed methods research. Accordingly, quantitative and qualitative approaches at all stages of the research interact with each other in a dynamic, dependent and repetitive manner (Tashakkori and Teddlie, 2003; Creamer, 2020). In this sense, it is aimed to base the study paradigmatically on pragmatism and accordingly, it was aimed to develop a psychoeducational application to intervene in the problem of school burnout, which is frequently encountered in schools, to test its effectiveness and to explain the experiences in the process. In this context, the research process is as in Figure 1, and the steps followed in this process are as in Figure 2.

This research has *development, complementarity and extension rationales* among the mixed method research rationales. Rationale for *development* (use of findings obtained from a method in other stages and dimensions of the research) to form the content of the psychoeducation program; the *complementarity* rationale for the qualitative data collected during and after the experimental application and the quantitative findings obtained from the experimental dimension to detail and explain by the using findings obtained from the qualitative method; and the rationale for *expanding* the research using different research methods was used (Greene et al., 1989). According to Mason (2006), a qualitatively driven mixed methods approach has enormous potential for new ways to understand the complexity and contexts of social experiences. Therefore, in this research, a psychoeducational program was developed by referring to the views of students who experienced this phenomenon and the review of the literature in order to reveal the nature of school burnout. An experimental process with pre-test post-test control group was conducted to test the effectiveness of the developed program. Meanwhile, students’ evaluations in the experimental group for each session were obtained. With the completion of the applications, interviews were done with the students in the experimental group to explain their experiences in the 9-week psychoeducation process and to explain the findings obtained as a result of the statistical analysis of the pre-test and post-test applications. It can be said that the internal and external validity, credibility, transferability and verifiability of the findings obtained from the study are high, depending on conducting the study with a fully integrated mixed methods research (Mertens, 2019). Since the mixing points in fully integrated mixed methods studies should be clearly stated (Creamer, 2020), the mixing points of this study are as follows:

- Mixing in the writing of the basic research problem: “*How do the data of the documents and interviews obtained from secondary school students help explain the results of the intervention trial aimed at developing a psychoeducational program and testing the school burnout of secondary school students?*,”
- Mixing in determining the research design: *the research design was determined as a fully integrated mixed methods research, and repetitive and interactive quantitative and qualitative approaches were preferred in the process.*
- Mixing in data collection process: *Mixing was done during experimental application during data collection process.*
- Mixing in the sampling process: *Nested sampling method was used as the sampling method and qualitative data were collected from the group in which quantitative data were collected.*
- Mixing in the data analysis: *During the analysis, both qualitative data and quantitative data were mixed with each other and the analysis was completed.*
- Mixing in the interpretation and discussion: *Mixing was done after the analysis of the quantitative data of the experimental process and the qualitative data collected during and after the experimental process, and all findings were interpreted, integrated and discussed.*

In addition, the research was carried out with a mixed methods research priority rather than quantitative or qualitative method. Both a *concurrent* and a *sequential* process can be mentioned in data collection and analysis. There is a *concurrent* process in the development of the content of the psychoeducation program, a *sequential* process during the experimental application and a *concurrent* process after the process.

### Study Group, Participants and Data Sources of the Study

The target population of this study is secondary school students who study in Erzurum province and experience school burnout, and the reason for choosing the adolescent students in the study group is to carry out an intervention process to prevent the negative effects of school burnout in the early stages. The research was initiated with purposeful sampling and interviews were conducted with 14 students who were determined to have school burnout before the psychoeducation application. In order to develop the content of psychoeducation, depending on the inclusion criteria, 8 theses and 44 articles are data sources for the document review step of the research. In the experimental process, nested sampling method, one of the mixed methods sampling strategies, was used (Creamer, 2020). Participants and groups were determined based on randomness. In this context, each of the experimental and control groups of the study consisted of 15 students, 8 boys and 7 girls, attending 7th grade. In addition, participants were selected among these students for the session evaluation forms collected during the experimental application (13 students) and the semi-structured interviews conducted after the experimental application (14 students).

### The Validity and Reliability of the Research

One of the factors threatening internal validity in experimental studies is *sampling error*. This was prevented by the *randomness* in both the selection of the participants and the assignment of the groups. In addition, studies were conducted in two different schools to prevent the *interaction* of experimental and control groups. Considering the possibility of *subject loss*, the experimental and control groups were composed of 15 students each. To minimize the effect of participants’ attitudes, which is another factor affecting internal validity, the principle of volunteerism was taken into consideration and the consent of both the family and the student for voluntary participation in the application was obtained. For the *statistical validity* of the study, the parametric test conditions of the collected data were examined and ANCOVA was done to determine the effectiveness of the intervention. The measures taken to increase the *construct validity* can be demonstrated by diversifying the theoretical and psychological framework of the psychoeducation program (McMillan and Schumacher, 2010). In addition, methods and data were diversified in order to control the *credibility* in qualitative processes. In addition, experiment fidelity was ensured in order to increase the reliability of the study, and any item addition or removal that could affect the structure of the measurement tools used was not performed.

### Data Collection Tools

#### Document Review Protocol

In the analysis of the studies determined to be suitable in line with the inclusion criteria determined within the scope of the study, the protocol developed by the researchers was used to include the author information of the studies, the year of publication, the status of the study as a thesis/article, the key concepts of the study, and the basic finding of the study and the process was carried out systematically by preventing possible data loss.

#### School Burnout Scale

The School Burnout Scale developed by Salmela-Aro et al. (2009a) was adapted into Turkish by Seçer et al. (2013) in order to determine the school burnout of students between the ages of 10–18. Exploratory and confirmatory factor analyzes were conducted to verify the latent structure of the scale and the structure expressed in the original form. A three-factor structure that explains 66.85% of the total variance was obtained as a result of the exploratory factor analysis. As a result of the confirmatory factor analysis, RMSEA = 0.060, RMR = 0.042, NFI = 0.97, NNFI = 0.97, CFI = 0.98, IFI = 0.98, RFI = 0.95, AGFI = 0.92, GFI = 0.96 compliance values were reached and it was stated that these values were significant. It is a self-reported measurement tool in a 4-point Likert structure consisting of 9 items and 3 sub-dimensions. As an example of the items in the scale, “Lessons have started to seem meaningless lately”; “I feel that I am getting bored with the lessons” can be given. The internal consistency coefficient was 0.75, the split-half reliability coefficient was 0.72, and the test–retest reliability coefficients were 0.84 for the emotional exhaustion dimension, 0.83 for the depersonalization sub-dimension, and 0.83 for the low sense of personal accomplishment.

#### Semi-structured Interview Protocol

During the research process, two different semi-structured interview protocols were developed by the researcher. While the first was aimed at revealing the perceptions and definitions of secondary school students regarding school burnout before the intervention program. The interview form aims to determine the views and perceptions of students who experience school burnout. In this context, the researchers prepared an interview form consisting of 8 questions. As an example of the questions in the form, “Have you had difficulty in fulfilling your duties and responsibilities (homework, project, etc.) lately? If so, what do you attribute it to?, Have you had any recent changes in your interest in school? If yes, to what do you attribute this?, How do you evaluate your recent study time? Do you think you spend enough time to have fun and rest apart from this time?” is located. The interviews held within this scope lasted approximately 45 min.

The second semi-structured interview protocol was used to evaluate the intervention program and explain the quantitative findings after the intervention program was implemented. In the development of both forms, necessary corrections were made by obtaining the opinions of both experts and peers. The interview form was used after the experimental procedure was completed. In this context, an interview form that was created to help explain the findings obtained after the quantitative analyzes were completed in accordance with the mixed method was used. An example question “Can you explain the reflection of the group experience you participated in your life?” It was used in individual interviews, which lasted an average of 10 min.

#### Session Evaluation Protocol

The session evaluation protocol used during the experimental process was also developed by the researcher, and the opinions of the students in the experimental group about the sessions were reached in writing. While developing the session evaluation protocol, both peer and expert opinions were consulted and when a consensus was reached, the form was finalized. In the session evaluation form, which consists of 5 questions, questions such as “How would you describe your general view of the session?, How did you feel” were asked and the answers were received in written form within an average of 10 min.

### Process and Application

In order to carry out the experimental process, the School Burnout scale was applied to 8^th^ grade students in two determined schools and the data obtained were analyzed. The opinions of the branch teachers were also taken for the students who were determined to have school burnout through the analysis. The families of the students, who volunteered to participate in the psychoeducation program, were contacted and informed with an informed consent form, and the necessary permissions were obtained from their families. With the determination of the experimental and control groups, a 9-week psychoeducation training was carried out in the experimental group, and guidance activities that were in the curriculum and not related to the dependent variable were applied to the control group. During the process, the students in the experimental group filled in the session evaluation protocols, and when the process was completed, semi-structured interviews were conducted with the post-test. Both a *concurrent* and a *sequential* process can be mentioned in data analysis process. In the first stage, both semi-structured interview data and document review data were analyzed with qualitative data analysis and a psychoeducation training program was developed. Measurements were made with the school burnout scale before and after the experimental application. SPSS 22.0 Package software was used in the analysis of the quantitative data obtained within this scope. Homogeneity, normality and extreme data analysis were performed to determine whether the data met the parametric test conditions. After determining that the parametric conditions were met, firstly independent samples t-test was conducted to determine whether there was a significant difference in terms of the dependent variable between the control and experimental groups. Secondly, Single-Factor Analysis of Covariance (ANCOVA) was done to determine whether there was a significant difference between the post-test scores of the experimental and control groups after the experimental procedure. Content analysis was used for the depth analysis of the qualitative data obtained from the session evaluation protocols during the experimental process and from the interviews after the experimental process and to examine the relationships in more detail (Yıldırım and Şimşek, 2016). In general, the integration of the qualitative data collected in order to develop the psychoeducation content, and the mixing of the quantitative and qualitative data related to the experimental process were done.

The analysis of the pre-test in Table 1 and post-tests in Table 2 and evaluation of the experimental and control groups in terms of pre-test in Table 3 are presented.

**Table 1:** Pre-test descriptive statistics and skewness-kurtosis values of experimental and control groups on emotional exhaustion, depersonalization, low achievement perception, total burnout score.

Group		*N*	Median	Mean	Sd	Skewness	Kurtosis
Experiment	Emotional exhaustion	15	12.00	11.8	1.08	−1.10	1.94
	Depersonalization	15	10.00	10.13	0.99	−0.299	0.617
	Low personal achievement perceptions	15	7.00	6.80	1.14	−0.538	−1.05
	School burnout total score	15	30.00	30.06	2.25	−0.616	0.733
Control	Emotional exhaustion	15	11.00	10.66	0.617	0.312	−0.404
	Depersonalization	15	10.00	10.26	1.27	−0.103	−1.11
	Low personal achievement perceptions	15	7.00	6.66	1.17	−0.158	−1.47
	School burnout total score	15	32.00	32.00	1.30	−0.220	−0.628

**Table 2:** Post-test descriptive statistics and skewness-kurtosis values of experimental and control groups on emotional exhaustion, depersonalization, low achievement perception, total burnout score.

Group		*N*	Median	Mean	Sd	Skewness	Kurtosis
Experiment	Emotional exhaustion	15	8.00	8.13	1.50	−0.114	−1.30
	Depersonalization	15	7.00	7.66	0.816	0.740	−1.02
	Low personal achievement perceptions	15	4.00	4.00	0.654	0.000	−0.179
	School burnout total score	15	20.00	19.80	1.56	0.508	−0.534
Control	Emotional exhaustion	15	11.00	11.06	1.22	0.127	−1.03
	Depersonalization	15	10.00	10.33	0.975	0.276	−0.646
	Low personal achievement perceptions	15	7.00	6.60	0.633	0.547	−0.385
	School burnout total score	15	28.00	28.00	1.41	0.175	−1.35

**Table 3:** Examination of school burnout pre-test scores according to experimental and control groups.

	Group	*n*	*X*	*SD*	*t*	*P*
Pre-test scores	Experiment	15	30.06	2.25		
	Control	15	32.00	1.30	−2.875	0.008

Tables 1 and 2 show the descriptive statistics and Skewness Kurtosis values regarding the pre-test and post-test applications.

When Table 3 is examined, the pre-test scores of the experimental and control groups differ significantly [*t*_(28)_ = −2.875, *p* < 0.05]. Accordingly, it can be said that the experimental and control groups are not equal in terms of pre-test scores. In order to prevent this situation, which is thought to affect the internal validity of the research, from being a source of problems, Single-Factor Analysis of Covariance was used, which allows the analysis of the change in the post-test by keeping the pre-test scores under control.

### Development of the Psychoeducation Program

In this study, a systematic psychoeducation program based on cognitive-behavioral therapy was developed to reduce the symptoms of school burnout of secondary school students. Cognitive-behavioral therapy is a therapy approach in which cognitive and behavioral techniques are used to develop an awareness of distorted thoughts and replace them with more functional thoughts (Sharf, 2015). In the development of this program, in the first stage, it was tried to determine the opinions and perceptions of secondary school students about school burnout by interview method in order to reveal the present symptoms of school burnout. The content of the psychoeducation program developed by combining the information obtained from the interview method with the themes emerging from the document analysis was determined. Briefly, in the intervention program design process, a literature review was first conducted for the conceptual framework of school burnout. At the same time, interviews were conducted with students who were determined to show symptoms of school burnout. Findings from these two data sources were integrated. Cognitive-behavioral therapy is based on a therapeutic approach. In this context, the agenda and content were determined for each session. The final version of the program was given after the opinions of the field experts. The intervention process lasted for 9 weeks and there was no loss of subjects in the process.

As a result of the phenomenological study conducted to determine the content of psychoeducation to be used in the experimental stage, participants’ perceptions of school burnout were grouped under three themes: *factors that predispose to experiencing school burnout, symptoms of school burnout* and *protective factors for school burnout*. The reasons that predispose to experiencing school burnout are discussed in three groups as *social factors, factors related to school* and *factors related to lessons*. Symptoms indicating that they experience school burnout were discussed in two sub-themes: *school-related symptoms* and *personal symptoms*. Participants’ views on the factors that protect them from school burnout were discussed in four groups: *having social support resources, ensuring school-family cooperation, enriching the learning environment with various tools* and *teachers’ positive attitudes and approaches*.

During the document review process inclusion criteria;

- Published in the last five years.
- Have open access.
- The publication language is Turkish or English.
- Publications in the Ulakbim index and National Thesis Center.
- Having the phrase school burnout in the title of the publication.
- Being accessible with “school burnout” as a key concept.

In order to develop the content of psychoeducation, 8 theses and 44 articles including theses published in the Council of Higher Education National Thesis Center in the last five years, which are open to access and articles published in journals that are included in the ULAKBIM index and have web archives and the term “school burnout” in the title of these publications and can be accessed with a keyword were determined as the data sources of the document analysis dimension of this study.

Descriptive information about the studies examined as data sources for the study is summarized in Table 4.

**Table 4:** Data sources of document analysis.

Author and publication year	Publication type	Keywords	Result
Burhan Çapri, Gülriz Yedigöz Sönmez (2013)	Article	High school students, burnout, psychological symptoms, attachment styles	As a result of the research, it was found that there were predictive relationships between burnout sub-dimensions, attachment styles and psychological symptoms.
Gülriz Yedigöz Sönmez, Burhan Çapri (2013)	Article	Student burnout, stress coping program, high school students	As a result of the research, it was found that the Coping with Stress Program had a permanent effect on reducing the scores of the students in the experimental group regarding the dimensions of emotional exhaustion and depersonalization.
İsmail Seçer, Sultanberk Halmatov, Fatih Veyis, Bünyamin Ates (2013)	Article	Burnout, school burnout, reliability, validity, scale adaptation	As a result of the research, it was found that the school burnout scale is a reliable and valid measurement tool for the Turkish sample.
Serap Kara (2014)	Master Thesis	Burnout, school burnout, psychological well-being, academic success	Result of the research, negative significant relationships were found between psychological well-being and school burnout.
Zekeriya Çam, Kaan Zülfikar Deniz, Arzu Kurnaz (2014)	Article	School burnout, perceived social support, perfectionism, stress, structural equality model	As a result of the research, it was found that social support caused a decrease in school burnout scores; It was concluded that stress increased in perfectionist individuals, increased stress caused school burnout, and school burnout brought depersonalization with it.
Pınar Yeni Palabıyık (2014)	Article	Burnout, language proficiency, emotion, depersonalization	As a result of the research, no relationship was found between school burnout and English proficiency levels.
Eyyüp Akıl, Taha Yazar (2014)	Article	Prep class, student burnout, burnout scale	As a result of the research, it was found that school burnout differed significantly according to whether or not to come to the department voluntarily.
Filiz Bilge, Meliha Tuzgöl Dost, Bayram Çetin (2014)	Article	Academic success, working habit, high school students, school engagement, burnout, belief in self-efficacy	According to the findings obtained as a result of the research, if the students’ self-efficacy belief is low, the level of burnout is high.
Kemal Avara (2015)	Master Thesis	Academic motivation, academic self-efficacy, career decision competence expectation, school burnout	According to the findings obtained as a result of the research, it was found that career decision competence expectation, academic self-efficacy and school burnout significantly predicted academic motivation. In addition, it was found that there is a negative and mediator relationship between school burnout and academic motivation.
Birkan Büyükarıkan, Ulukan Büyükarıkan (2015)	Article	Burnout, maslach burnout scale, science, graduate. education	As a result of the research, it was found that the dimension of emotional exhaustion was higher in graduate students who were in the course period.
İsmail Seçer (2015)	Article	School burnout, psychological adjustment, stress, structural equation modeling	As a result of the research, it was found that psychological maladjustment had indirect and direct effects on emotional exhaustion, depersonalization, and low personal achievement perceptions and predicted school burnout significantly in total.
Yalçın Özdemir (2015)	Article	School engagement, academic motivation, school burnout	As a result of the research, there were negative significant relationships between academic motivation and school engagement and school burnout, while a positive significant relationship was found between the time allocated for homework and school burnout.
Hakan Acar, Mehmet Ali Çakır (2015)	Article	Secondary education, school burnout, burnout	As a result of the research, it was found that the school burnout levels of secondary school students differed significantly according to their housing status, gender, monthly income of their families, and the field they studied.
Lokman Koçak (2016)	Master Thesis	Burnout, school burnout, depression	As a result of the research, it was found that there was a significant and positive correlation between anxiety and depression and school burnout; anxiety and depression were found to be significantly predicted by school burnout.
Hatice Özgen (2016)	Master Thesis	University students, school burnout, need for psychological help	As a result of the research, a significant and positive relationship was found between the need for psychological help and school burnout.
Servet Atik (2016)	Doctoral Dissertation	High school students, academic achievement, trust in teacher, attitude towards school, school burnout, alienation from school, structural equation modeling	As a result of the research, it was found that distrust in teachers affects school burnout negatively and directly; it has been revealed that school burnout is positively affected by alienation from school, and negatively and significantly affected by students’ attitudes towards school. In addition, it was found that school burnout had a negative effect on their academic achievement.
Medina Akpınar (2016)	Master Thesis	School burnout, academic stress, subjective well-being, mediation relationship	As a result of the research, it was found that school burnout has a full mediator role between academic stress and subjective well-being.
Zeynep Banu Gündüz (2016)	Master Thesis	Parental attitude, self-esteem, school burnout, secondary school students	As a result of the research, it was found that the students who perceived their parents’ attitudes as authoritarian and protective had a decreased self-esteem and increased school burnout levels, while the students who perceived their parents’ attitudes as democratic increased their self-esteem and their school burnout levels decreased.
Şahin Kapıkıran, Metin Yaşar, Necla Acun Kapıkıran (2016)	Article	Self-esteem, school burnout, self-regulation, high school	As a result of the research, it was found that self-regulation has both an indirect and a direct role in the relationship between school burnout and self-esteem.
Bünyamin Ateş (2016)	Article	Solution focused psychological counseling, school, school burnout, high school students	According to the findings obtained from the research, solution-oriented group counseling was effective in the school burnout of high school students.
Ayşe Aypay, Emine Durmuş, Eren Can Aybek (2016)	Article	Peer bullying, school burnout, parental monitoring, victim	As a result of the research, it was found that the scores of being a victim of verbal and relational bullying as the level of burnout caused by family increases, of being a victim of verbal bullying as the level of school burnout caused by school activities increases, while the scores of being a victim of physical bullying decrease as the level of burnout caused by loss of interest in school increases.
Gufran Gündoğmuş (2017)	Master Thesis	Loneliness, attitude, parental attitude, burnout, school burnout, adolescence	As a result of the research, it was observed that loneliness was significantly predicted by burnout caused by family, loss of interest in school, inadequacy at school, and democratic parental attitude.
Hakan Sarıçam, İsmail Çelik, Halis Sakız (2017)	Article	Metacognition, education stress, school burnout, adolescents	As a result of the research, it was determined that metacognitive awareness had a mediator role in the relationship between educational stress and school burnout, and school burnout was strongly predicted by educational stress.
Birsen Şahan, Baki Duy (2017)	Article	School burnout, self-efficacy, school attachment, friend support, secondary school	As a result of the research, it was found that school burnout was predicted significantly by the teacher attachment variable.
Meva Demir, Adem Peker (2017)	Article	Motivation, burnout, structural equation model	The main finding of the study is that school burnout is significantly predicted by motivational determination.
Meva Demir, Başaran Gençdoğan (2017)	Article	Burnout, exam anxiety, academic success	The main finding reached as a result of the research is that academic achievement and test anxiety have a predictive role in school burnout.
Gürcan Şeker, Yasemin Yavuzer (2017)	Article	School burnout, academic locus of control, adolescents	As a result of the research, there was a positive and moderate relationship between school burnout and external locus of control; it was found that there is a negative and moderate relationship with the internal locus of control.
Emine Durmuş, Ayşe Aypay, Eren Can Aybek (2017)	Article	Parental monitoring, positive school climate, school burnout, high school, model testing	As a result of the research, it was determined that there is a negative relationship between parental monitoring and school climate, and school burnout.
Ayşe Aypay (2017)	Article	School burnout, reward addiction, academic context, high school, student, social	As a result of the research, it was found that reward commitment predicted school burnout.
Aygen Çakmak, Hande Şahin (2017)	Article	Secondary school students, school burnout, social relationship elements	As a result of the research, it was found that the school burnout levels of the students differed significantly according to the age of the parents, socioeconomic status, education level of the parents, class, and gender.
Pervin Nedim Bal, Casim Kaya (2017)	Article	School burnout, solution focused group counseling, 6th grade students	As a result of the research, it has been found that solution-oriented group psychological counseling is effective in the intervention of school burnout.
Yusuf Çelik, Ahmet Üstün (2017)	Article	Burnout, burnout, depersonalization, competence, senior university students	As a result of the research, it was found that the school burnout scores did not differ significantly in terms of the regions where the students came from and the gender variable, and the school burnout scores differed significantly in terms of the department they studied. In addition, it was determined that the family socioeconomic level variable showed a significant difference only in the dimension of depersonalization.
Nuray Taştan, Rıza Gökler (2017)	Article	Cyberbullying, school burnout, student	As a result of the research, it was found that there is a moderate and positive relationship between school burnout and cyberbullying.
Aylin Arıcı, Taner Artan, Merve Çiçek, Yalçın Özbek, Doğac Niyazi Özüçelik (2017)	Article	School social work, creative drama, life satisfaction, school burnout	As a result of the research, it was found that creative drama activities were effective in reducing school burnout.
Dilara Saka, Sabahat Burak (2018)	Article	Music education, music lesson downloads, school burnout	As a result of the research, it was found that there is a negative relationship between students’ school burnout and music lesson loading levels.
Ayşe Aypay (2018)	Article	Sensitivity to punishment, school burnout, sense of school, sense of belonging to school	As a result of the research, it was found that there is a positive relationship between the students’ punishment sensitivity sub-dimensions and school burnout levels.
Zeynep Banu Gündüz, Arzu Özyürek (2018)	Article	School burnout, parental attitude, high school students	As a result of the research, it was found that students who perceive parental attitudes as protective and authoritative have higher levels of school burnout, while students who perceive it as democratic have lower levels of school burnout.
Şule Polat, Murat Özdemir (2018)	Article	Educational stress school burnout school alienation secondary school students	As a result of the research, significant relationships were found between school burnout, school alienation, and educational stress, and it was found that school burnout and educational stress predicted school alienation significantly.
Gülcihan Arkan, Yaprak Sarıgöl Ordin, Meryem Öztürk (2018)	Article	Professional values, ethics, burnout, nursing students	As a result of the research, it was found that as the burnout levels of the nursing department students increased, their professional values decreased.
Mehmet Boyacı, Mehmet Buğra Özhan (2018)	Article	School burnout, hope, family relations, structural equality model school guidance	As a result of the study, it was found that there was a negative relationship between hope and supportive family relationships and school burnout, while a positive relationship was found between disruptive family relationships and school burnout.

As a result of the analysis variables that were frequently addressed in school burnout studies between 2013 and 2018 were grouped under two themes: *variables related to psychological experiences and variables related to academic experiences*. Secondly, these variables related to both academic and psychological processes were categorized as negatively and positively related variables. Thirdly, it was discussed that school burnout has a predictive role for variables that are found to be associated with school burnout and which variables predict school burnout.

### Integration of the Findings Obtained From the Interview Method and Document Analysis

From the interviews and the documents examined, it is found that the variables that play a role in experiencing school burnout are the effect of friends, teachers and family, the loss/lack of motivation, the intensity of the exams and the exam anxiety, academic motivation, alienation from school, depression, anxiety, academic stress, psychological well-being, school engagement, trust in teachers, ensuring school-family cooperation, teachers’ positive attitudes and approaches, having a source of social support, academic self-efficacy belief, and self-regulation skills. In the findings obtained from the interviews, it has been determined that social support, teachers’ positive attitudes toward students, ignoring students by teachers and the family’s excessive expectations from the students, comparison in peer groups, intensity and complexity of the lessons, inability to use time efficiently, lack of motivation, doubt about the student’s ability and potential, procrastination, forgetfulness, indifference, avoiding taking responsibility, enriching the learning environment with various tools and ensuring school-family cooperation have come to the fore and while social support, trust in teachers, competence beliefs, attitude toward school, and family’s attitude coincide with document analysis, it was seen that psychological symptoms, need for psychological help, attachment styles, academic motivation, school engagement, metacognition, self-regulation, subjective well-being, depression, anxiety and educational stress were obtained from different data sources as different codes. Therefore, when the findings obtained from two different data sources are integrated, the main elements that make up the content of the relevant intervention program are summarized as follows.

School burnout psychoeducation program session contents (Table 5).

**Table 5:** The contents of the psychoeducation program.

Session	Content
1. Session	*Meeting (Structuring) Session*
2. Session	*Expressing their academic life and analyzing these experiences in terms of school burnout components*
3. Session	*Students’ reactions to the situations in the second session and an analysis of these responses in terms of functionality*
4. Session	*Handling the phenomenon of school burnout with a cognitive model*
5. Session	*Focusing on the perception of low personal achievement perceptions, addressing the processes of doubting about their ability and potential and developing self-incriminating attitudes.*
6. Session	*Focusing on forgetfulness, fatigue, failure to fulfill school-related duties and responsibilities, escape from duties and responsibilities, and emotional exhaustion*
7. Session	*Focus on the concepts of indifference to courses, academic procrastination, lack of motivation and loss of motivation, and the dimension of depersonalization.*
8. Session	*Focus on motivation, academic motivation, social support concepts and factors with protective roles*
9. Session	*Expressing the acquisitions gained during the group process, planning what can be done to protect and increase the acquisitions gained during the sessions after the group*

## Results

*Research Hypothesis:* Psychoeducation application developed based on CBT is an effective approach in reducing school burnout in secondary school students.

In the process of testing this hypothesis, three main dimensions of school burnout were examined, as suggested in the relevant literature and included in the measurement tool. In the analysis process, analysis of covariance was used, and the equivalence of regression tendencies was tested as the prerequisite criterion of the analysis. Accordingly, the equivalence of regression tendencies is for emotional exhaustion [*F*_(1,29)_ = 0.159, *p* = 0.693], depersonalization [*F*_(1,29)_ = 0.143, *p* = 0.708] and low personal accomplishment [*F*_(1,29)_ = 0.239, *p* = 0.629]. Considering that the criteria suggested for all three dimensions were met, the experimental and control groups were compared separately for the three sub-dimensions. In addition, considering that the research was designed with a mixed methods research, each finding was mixed by indicating those obtained from different data sources altogether.

Analysis results regarding the emotional exhaustion dimension are given in Table 6.

**Table 6:** Analysis results regarding emotional exhaustion.

Source	Sum of squares	*df*	Mean square	*F*	Sig.	*η^2^*
Corrected model	73.211	2	36.606	22.468	0.000	0.625
İntercept	1.072	1	1.072	0.658	0.424	0.024
**Emotional exhaustion pre-test**	**8.678**	**1**	**8.678**	**5.327**	**0.029**	**0.165**
Group	69.212	1	69.212	42.482	0.000	0.611
Error	43.989	27	1.629			
Total	2882.00	30				
Corrected total	117.20	29				

The results of the analysis regarding the effectiveness of the experimental procedure with bold lines are highlighted.

When Table 6 is examined, it can be said that the cognitive-behavioral therapy-based school burnout psychoeducation program applied to secondary school students provided a significant decrease on the emotional exhaustion scores of the students in the experimental group [*F*_(1,29)_ = 5.327, *p* = 0.029, *η*^2^ = 0.165]. Bonferroni test results showed that the results obtained in the post-test scores were in favor of the experimental group.

It has been observed that the findings from the session evaluation protocols obtained from the experimental application explain the result of the experimental process. The evaluations of the students participating in the school burnout psychoeducation application related to the reactions they show against the school burnout were categorized under two themes as functional reactions and dysfunctional reactions. An example of the code of emotional exhaustion in the dysfunctional reactions category is by *K11* “*I get bored, overwhelmed, worried, tired and sluggish. I cannot attend the lesson because of tiredness, I am constantly sleepy. As such, my homework piles up and I worry. Then, I regret*.” In addition, direct quotations about their functional responses to the emotional exhaustion dimension can be exemplified as by *K12* “*I learned about distortions, for example, I can notice the distortions I make during the day. I learned how to deal with these. I learned that when my reactions are not functional, they will continue. I started to be more sensitive and calmer to things*.”

Sample expressions taken from the interviews made after the implemented program are as by *K7* “*I was also telling my parents, for example, I was asking “Mom, I get bored in lessons, what’s the reason for this” and* etc. *They never took me seriously, but I told you, I poured my heart out and relaxed. It contributed in this way. I also learned motivation, my teacher, I learned intermediate beliefs, basic beliefs, my teacher, I learned distortions. In this way, my indifference and boredom towards the lessons decreased*” and by *K12* “*For example, I am calmer towards my teachers and friends, and I do not give adverse answers. I also do my homework without delay, even if I am tired, I will not delay. So, I see the effect,*” and explain the significant difference found in the emotional exhaustion dimension.

The analysis for the depersonalization dimension, another dimension of school burnout, is given in Table 7.

**Table 7:** Analysis results regarding depersonalization.

Source	Sum of squares	*df*	Mean square	*F*	Sig.	*η^2^*
Corrected model	61.527	2	30.764	57.392	0.000	0.810
İntercept	6.081	1	6.081	11.345	0.002	0.296
**Depersonalization pre-test**	**8.194**	**1**	**8.194**	**15.286**	**0.001**	**0.361**
Group	50.658	1	50.658	94.506	0.000	0.778
Error	14.473	27	0.536			
Total	2506.00	30				
Corrected total	76.00	29				

The results of the analysis regarding the effectiveness of the experimental procedure with bold lines are highlighted.

When Table 7 is examined, it can be said that cognitive-behavioral therapy-based school burnout psychoeducation program applied to secondary school students provided a significant decrease on the depersonalization levels of the students in the experimental group [*F*_(1,29)_ = 15.286, *p* = 0.001, *η*^2^ = 0.361]. Bonferroni test results showed that the results obtained in the post-test scores were in favor of the experimental group.

Qualitative findings obtained from the session evaluation protocols explain this. The evaluations of students participating in the school burnout psychoeducation program regarding their reactions to school burnout, a dysfunctional sample directly quoted from the depersonalization code by *K3* as *“The teachers do not call me by my name and they use bad words, I am just annoyed by this situation, just annoyed,”* functional responses for desensitization code by *K6 “It is challenging for me that my friends know all the answers, my teachers always set someone example, I am guilty about issues that are not related to me. When I undergo through these situations, I ask why they do it, I say I do not want them to do it,” K3 “I mean, I did not want to come to school anymore for a moment, I even thought if I should quit. But I realized I had goals. I realized that to achieve these goals, I had to be in school. When I started these sessions with you, I realized that it is more necessary for me to reach these goals. Well, while I was studying, I used to think “I should not study, why am I studying? What is the point? but now I realized that I have to study.”*

Sample expressions taken from the interviews made after the program are by *K1 “For example, my teacher, I understood that I should not upset myself with what others said. Also, my teacher, for example, I learned some distortions, what caused them when I made things too big or too small,” “I can say that this application helped me to know myself. I learned about cognitive distortions. For example, I use it when deciding how to behave when something happens between me and someone,”* and it explains the significant difference found in the depersonalization dimension.

The analysis for the low personal accomplishment dimension, another dimension of school burnout, is given in Table 8.

**Table 8:** Analysis results regarding perception of low personal achievement perceptions.

Source	Sum of squares	*df*	Mean square	*F*	Sig.	*η^2^*
Corrected model	53.350	2	26.675	80.474	0.000	0.856
İntercept	10.008	1	10.008	30.194	0.000	0.528
**Perception of low personal achievement perceptions pre-test**	**2.650**	**1**	**2.650**	**7.995**	**0.009**	**0.228**
Group	51.904	1	51.904	156.585	0.000	0.853
Error	8.950	27	0.331			
Total	905.000	30				
Corrected total	62.300	29				

The results of the analysis regarding the effectiveness of the experimental procedure with bold lines are highlighted.

When Table 8 is examined, it can be said that cognitive-behavioral therapy-based school burnout psychoeducation program applied to secondary school students provided a significant decrease on the low personal achievement perceptions of the students in the experimental group [*F*_(1,29)_ = 7.995, *p* = 0.009, *η*^2^ = 0.228]. Bonferroni test results showed that the results obtained in the post-test scores were in favor of the experimental group.

In the session evaluation protocols, the direct quotation regarding the perception of low personal accomplishment dysfunctional reactions code is *K1 “The question of whether I can do it causes me anxiety or I’m afraid if I get lower marks. This time, my self-confidence decreases, and I do not want to talk, I do not want to listen.”* For low personal accomplishment code, functional responses can be exemplified as *K6 “I constantly fear whether I will fall out of favor with the teachers. I become much more ambitious in case someone does it better than me. Telling myself that I have to compete with myself and have confidence,” K8 “I get anxious when I get a low grade in a course. I found out that I was making an exaggeration. I realized that I was not failing, I was hardworking. I developed the alternative idea of “you are already successful, you got low in a lesson” and I was happy with this idea.” K7 “I wish I could be successful in all subjects; I tell myself that I should do, I should study, and I realized that these were necessity statements or all-or-nothing thoughts,” K11 “I have a lot of anxiety about the lessons, I have no self-confidence. I experience sadness and pessimism by making things disasters to me,” K1 “My teacher, after trying to do more in the exams and if I get higher marks, I tell myself that OK, K1, you did this, you can do it in the future. It has been useful for me.”*

Sample expressions taken from the interviews made after the program implemented are *K7 “I believe more now that I can do things,” K8 “I realized that I would never break my mood and move on, consistently adhere to a plan and concentrate, and if I have something in my mind, I will achieve that. So, with these sessions, my belief that I can do something for myself increased,” K6 “My goal was to get higher marks from the exams. I think I achieved this. I used to have 22 wrong answers, but now I’ve pulled it into the range I wanted. And as such, my belief in going to a science high school increased. So I can say that this program has an impact on my study,”* and this explains the significant difference found in the low personal accomplishment dimension.

## Discussion

In this research, a psychoeducational program for children with high symptoms of school burnout was developed and its effectiveness was examined. In the process of both developing the intervention program and examining its effectiveness, data triangulation was done in line with the mixed methods research approach. In this sense, the process started with a qualitatively oriented approach. In this context, the structure of school burnout was determined based on document analysis and phenomenology patterns and the intervention program was shaped. In the second phase of this process, quantitative and qualitative data collection processes were carried out in order to test the effectiveness of the developed intervention program. In the last stage, all the findings obtained were mixed and interpreted in order to provide high validity and reliability.

In this context, the experimental process carried out regarding the effectiveness of the school burnout intervention program developed based on CBT is the quantitative dimension of the research. The findings obtained from this quantitative dimension reveal that the intervention program is an effective approach in reducing the symptoms of *emotional exhaustion, depersonalization* and *low personal accomplishment* (Salmela-Aro et al., 2009a,b), which are three main dimensions of school burnout. This finding is consistent with the limited number of research results that include interventions for school burnout in the literature (Salanova et al., 2010; Anggreini et al., 2019; Susanti et al., 2019). However, with the thought that quantitative approaches focusing on statistical validity as a requirement of positivist approaches will constitute an important limitation. it was aimed to strengthen the validity by explaining and expanding the findings obtained by trying to learn how the students’ experiences in the process shaped and to include these in the analysis (Mertens, 2019; Creamer, 2020). In this respect, it was determined that the analyzes carried out in two different qualitative processes, during and after the experimental application, generally explained the quantitative results and supported the findings regarding the effectiveness of the intervention program implemented.

Quantitative findings indicate that symptoms related to burnout significantly decrease after the intervention and the document analyzes obtained during the experimental application show that the cognitive distortion processes, which constitute the main focus of CBT, are understood by the students sufficiently and that the cognitive model is used effectively (K11, K1, K7) in the development of effective coping approaches. Similarly, the findings obtained from phenomenological interviews (K7, K12) can be evaluated as evidence that the cognitive model is used effectively both in the academic process and in daily life (K3). In the findings obtained from the interview processes after the application, it was determined that the students’ awareness of cognitive distortions that prepared the ground for school burnout increased (K7, K11), and the participants used expressions (K1, K3, K6, K7, K12) showing that the practice they participated in was effective in developing healthy cognitions and responses. In addition, it was found that there were some efforts to apply the cognitive model taught during the application in their daily lives (K6, K12) and it was concluded that these findings support and confirm the quantitative results.

In addition to the fact that quantitative findings show that there is a significant difference between pre-test and post-test measures, it is also possible to see the change in children more subjectively through qualitative findings (Mason, 2006). In this sense, it is understood that with the obtained qualitative findings, students frequently use overgeneralization, personalization and selective abstraction distortions and learned to distinguish their reactions as functional and dysfunctional reactions. Increasing children’s awareness of cognitive distortions and functional coping approaches is an important achievement of intervention practice and is expected to positively affect not only the school life but also the daily life.

In this context, as a result of the study conducted by Farina et al. (2020), it was found that the level of satisfaction with peer and adult relationships at school mediator the relationship between empathy skills (affective and cognitive) and school burnout as a risk factor. It has been evaluated that the intense use of the affective dimension of empathy is a risk factor for emotional exhaustion. The qualitative findings of the study were also found to be compatible with the intense emphatic concerns of the students, as well as feeling unable to cope with school-related responsibilities or feeling overwhelmed by the weight of these tasks (Wagaman et al., 2015; Bloom, 2017). In addition, studies have shown that students become overwhelmed with school and their work due to the decrease in their evaluations of the school and their feelings of emotional attachment to the school, as a result of seeing the school as less valuable, and a result, they experience school burnout. It has been found to be effective (Wang et al., 2015). In the PISA 2012 (Organisation for Economic Co-operation and Development, 2013) results, it was reported that Korean students with the highest academic performance level were also the most unhappy students with the effect of decreased motivation and school engagement in the transition to high school education. In this context, the finding obtained from the study that there are exams in the transition to the next education level and that both parents and teachers have expectations above their abilities and skills is important in terms of experiencing burnout, parallels the report.

In addition, students’ perfectionist tendencies and inadequacy of coping skills were found to be closely related to school burnout (Lau et al., 2020). A meta-analysis study by Kim et al. (2018) revealed that social support, which is closely related to the depersonalization dimension of school burnout, is effective in reducing school burnout. In this study, it was found that the perceived social support from parents, teachers, and peers helped students to stay away from the school context and develop a sense of belonging.

Social support has a mediator role between school burnout and academic self-efficacy (Aslan, 2018), and in the study conducted by Ulaş and Seçer (2018), academic self-efficacy has a mediator role in the relationship between school burnout and psychological maladjustment. Within the scope of this study, with the analysis of the goals that the students set for themselves in the content of the psychoeducation program, it was seen that the majority of the students had goals to improve their interpersonal relations and communication skills. This situation was evaluated as an effort to get rid of the effects of the depersonalization dimension, which is characterized by the elimination of social exclusion or isolation.

Among the prevention and intervention studies on school burnout, (Salanova et al., 2010) applied a 4-month cognitive-behavioral approach based on social cognitive theory and aimed at reducing the burnout of university students and increasing their self-efficacy and performance. The main purpose of this program was expressed as minimizing students’ pre-exam anxiety levels and increasing their efficacy beliefs, and school burnout was handled indirectly, and the findings revealed that the practice was significantly effective on students’ school burnout. In the study conducted by Çapri and Yedigöz-Sönmez (2013), the effect of the stress-coping program on the burnout levels of high school students was examined and it was concluded that this program was significantly effective on the burnout of high school students. However, this study has additional academic factors such as lack of motivation, academic motivation, commitment to school, as well as the intensity of exams and test anxiety, the stress in general and academic stress in particular, at the point of experiencing school burnout obtained through interviews and document analysis before creating the psychoeducation program content. It has been found that psychosocial factors such as depression, anxiety, self-regulation, avoidance of responsibility, perceived parental attitudes, comparison within peer groups or lack of social support have an important place.

In this context, it has been evaluated that the studies conducted by Salanova et al. (2010) and Çapri and Yedigöz-Sönmez (2013) only focus on dealing with test anxiety and stress, creating a limitation in ensuring construct validity. İlbay (2014), in his study with university students, concluded that solution-based group counseling was significantly effective on school burnout experienced by students. In this study, as a result of the qualitative dimension carried out to create the psychoeducational content, it was seen that the role of the family in the burnout of students is undeniable. Salmela-Aro et al. (2009b), on the other hand, found that the burnout levels of parents would also reflect on adolescents. In this context, while the codes related to the family were found to be parental attitudes, unrealistic expectations of parents from their children and seeking perfection, and comparing children with their peers, it was seen that this situation led students to doubt their own abilities and potentials. In addition, it has been found that the cooperation of the school and the family is a factor that strengthens the position of children in terms of school burnout. Salmela-Aro et al. (2009b), it was seen that it was mostly fed by school-related factors (trust in the teacher, positive attitudes and approaches of the teacher, enriching the learning environment through various materials) compared to the qualitative findings obtained from this study. A group intervention based on Rational Emotive Behavior Therapy by Anggreini et al. (2019) focused on the academic dimensions of school burnout and it was found to be effective. It was found that the Hope Therapy application by Mahdavi et al. (2020) significantly reduced students’ burnout levels. Acceptance and Commitment Therapy Counseling and Mindfulness-based Cognitive Counseling are effective in reducing school burnout (Susanti et al., 2019; Ahmadi-Moghadam et al., 2020) find that it is effective in reducing school burnout, which is considered as a dimension of academic well-being. Found. It was found that the main theoretical views on which these practices are based are an extension of CBT, therefore, the problem in the intervention of school burnout stems from possible cognitive errors, and this situation can only be resolved by replacing these dysfunctional cognitions with functional cognitions.

### Study Limitations and Further Research

Although the research was conducted with an experimental design, which is strong in terms of cause-effect relationship, it has some limitations in terms of evaluation of the study group and the participants. The participants of the research were selected from the non-clinical group. Therefore, it is beneficial to conduct clinical trials as the results obtained cannot be generalized to clinical samples. In addition, in the context of multilevel approaches, it is thought that there is a need for practices that make parents and teachers a part of the intervention and data triangulation. The fact that the study was tested in a small group and only the school burnout scale was used as a measurement tool are limitations of the study. Finally, it is thought that the effectiveness of the program should be examined in other school levels other than the secondary school age group.

## Data Availability Statement

The raw data supporting the conclusions of this article will be made available by the authors, without undue reservation.

## Ethics Statement

The studies involving human participants were reviewed and approved by Atatürk University Educational Sciences Ethics Committee. Written informed consent to participate in this study was provided by the participants’ legal guardian/next of kin.

## Author Contributions

SU and İS worked together in the planning, practice, and reporting of this study. This study is based on a master’s thesis by SU and supervised by İS. Both authors contributed to the article and approved the submitted version.

## Conflict of Interest

The authors declare that the research was conducted in the absence of any commercial or financial relationships that could be construed as a potential conflict of interest.

## Publisher’s Note

All claims expressed in this article are solely those of the authors and do not necessarily represent those of their affiliated organizations, or those of the publisher, the editors and the reviewers. Any product that may be evaluated in this article, or claim that may be made by its manufacturer, is not guaranteed or endorsed by the publisher.
